# Bipolar Disorder Due to Traumatic Brain Injury: A Case Report

**DOI:** 10.7759/cureus.51292

**Published:** 2023-12-29

**Authors:** Yasir Altuwairqi

**Affiliations:** 1 Medicine/Psychiatry, Taif University, Taif, SAU

**Keywords:** bipolar disorder, frontal lobe, schizoaffective disorder, substance use disorder., traumatic brain injury (tbi)

## Abstract

I report an unusual case in Saudi Arabia of a 28-year-old man who had bipolar disorder due to a traumatic brain injury suffered 10 years previously. He had been evaluated and diagnosed with schizoaffective disorder as well as amphetamine and hash use disorder until recently, when the team noticed a poor response to treatment and the continuation of his cognitive features. After a reevaluation of the history and evidence of the brain lesions on the MRI, the diagnosis was changed to bipolar disorder due to a traumatic brain injury. The patient had shown a fair response to valproate and risperidone. This report emphasizes the significance of ruling out the medical factors contributing to the manifestation of any novel psychiatric symptom, necessitating greater attention to the account of cranial trauma and periods of unconsciousness. Psychiatrists should be aware of these overlooked cases and encourage colleagues in the field to maintain a high index of suspicion and to take a good relevant history of brain injury insults, especially when there are cognitive features and a poor response to medications. The patient exhibited symptoms of inattention, memory difficulties, reasoning deficits, and poor judgment, but he did not meet the criteria for a minor or major cognitive disorder.

## Introduction

Traumatic brain injury (TBI) can lead to a range of psychiatric consequences, which have a considerable influence on functional outcomes and the overall quality of life. The psychiatric disorders arising from TBI are diverse and can include mood disorders, anxiety, substance misuse, emotional lability, apathy, and an elevated risk of suicide. It can also lead to cognitive and behavioral deficits, including mild cognitive impairment and dementia [1]. A study focusing on the psychiatric outcomes of traumatic brain injury found that 12 months after injury, 31% of patients reported a psychiatric disorder, and 22% developed a psychiatric disorder they had never experienced before [2]. Aggressive and disinhibited behaviors frequently manifest in patients with TBI [3]. The neuropsychiatric consequences of TBI pose considerable difficulties for both patients and health-care practitioners [4]. It is important to accurately diagnose psychiatric presentations following TBI in order to administer effective treatment. Aggressive and disinhibited behaviors frequently manifest in patients with TBI [3]. The Diagnostic and Statistical Manual of Mental Disorders, Fifth Edition, Text Revision (DSM-5-TR) recognizes different diagnosis classifications and coding associated with traumatic brain injury according to the main psychiatric features and the associated symptoms [5].

The reported patient’s disorder had an organic cause that resulted in manic features with psychosis. So, the DSM diagnosis that fits best is bipolar and related disorder due to traumatic brain injury with manic features (F06.33).

The diagnosis of bipolar and related disorders due to another medical condition should be made instead of bipolar I disorder if the manic episodes are judged, based on history, laboratory findings, or physical examination, to be the direct physiological consequence of another medical condition, such as traumatic brain injury. When the predominating features are cognitive, then the differential diagnosis to be considered is major neurocognitive disorder due to traumatic brain injury with behavioral disturbance (F02.81). Behavioral disturbance refers to the presence of a cognitive disturbance that is accompanied by clinically significant behavioral disruptions (e.g., psychotic symptoms, mood disturbance, agitation, apathy, or other behavioral symptoms).

When psychosis emerges as the primary manifestation after a TBI, the DSM classifies the condition as a psychotic disorder due to traumatic brain injury with delusions (F06.2). The clinician’s best judgment remains important in applying these diagnoses.

Among the best-known medical conditions that can cause a bipolar manic or hypomanic condition are Cushing’s disease and multiple sclerosis, as well as stroke and traumatic brain injuries. The clinician must make a clinical judgment in these situations about whether the medical condition is causative, based on temporal sequence as well as the plausibility of a causal relationship.

TBI can be classified into mild, moderate, and severe forms depending on the extent of the injury, the length of unconsciousness, and the duration of post-traumatic amnesia. Symptoms associated with mild TBI include the presence of a headache, confusion, and memory complications. Moderate TBI symptoms are more severe and can include a persistent headache and seizures. Severe TBI is the most extreme and can result in unconsciousness or coma. The treatment for TBI is subject to variation according to the degree of severity of the presentation and encompasses medication, surgical intervention, and rehabilitation as potential therapeutic measures. The goal is to improve the patient’s ability to function at home and in society. TBI greatly affects one’s daily life, including work, relationships, and activities. Early intervention and rehabilitation yield favorable outcomes. To mitigate the risk of traumatic brain injury, preventive measures such as the use of helmets and seat belts play a crucial role [5].

## Case presentation

A 28-year-old Saudi, single, jobless man with a secondary school education was brought by his father to the emergency department, assisted by police; he reported that his son had become hyperactive and potentially verbally and physically aggressive over the previous week. Weeks before this, he had been gradually unable to sleep and had become talkative and impulsive. There were no reported injuries as a result of his violence, nor was self-harm observed. There were no suicidal or homicidal tendencies. The father, who was an impoverished historian, provided no more details but said that his son refused to cooperate with commands to go to the hospital until the police were contacted for help. The patient denied any psychotic or depressive features. He reported no drug history, significant medical history, or head injury. The gathered personal and family histories were unremarkable.

On mental state examination, the patient appeared to be an adult male with an average physique and normal grooming and self-hygiene. He was overfamiliar, verbally disinhibited, and hyperactive. His speech was coherent, with an increased tone and volume. The mood was elated, with a flight of ideas. The patient had delusions of reference and persecution but denied hallucinations. There were no homicidal or suicidal ideas. Cognitive functions were difficult to assess because he was uncooperative, but he appeared alert and oriented to time, place, and person. His attention and concentration were poor, and he was distractible. He had poor judgment and impaired insight.

The patient had been diagnosed with schizoaffective disorder. The differential diagnoses were bipolar I disorder, manic episodes with psychotic features, schizophrenia, bipolar disorder due to substance use, bipolar disorder due to another medical condition, and psychotic disorder due to another medical condition. His blood work on admission was normal (Table 1).

**Table 1:** Electrolytes, liver function test, renal function test, lipid profile, and complete blood count K: Potassium; NA: sodium; Cl: chloride; BUN: blood urea nitrogen; CREA: creatinine; ALP: alkaline phosphatase; ALT: alanine aminotransferase; ALB: albumin; TP: total protein; LDL low-density lipoprotein; HDL high-density lipoprotein; WBC: white blood cells; RBC: red blood cells; PLT: Platelet; HGB: hemoglobin.

Test	Result	Conclusion	Normal range
Random glucose	6.96	Normal	4.1–8 mmol/L
K	3.81	Normal	3.5–5.1 mmol/L
NA	139	Normal	136–145 mmol/L
CL	100.8	Normal	98–107 mmol/L
BUN	3.80	Normal	2.7–8.1 mmol/L
CREA	92	Normal	44–106 umol/L
ALP	111	Normal	35–129 U/L
ALT	14.88	Normal	5–41 U/L
ALB	40.9	Normal	35–50 q/L
TP	74	Normal	66–78 q/L
Triglycerides	0.84	Normal	0.1–1.7 mmol/L
Cholesterol	4.12	Normal	0.0–5.2 mmol/L
LDL	2.45	Normal	0.1–2.6 mmol/L
HDL	1.43	Normal	0.9–1.55 mmol/L
WBC	7.9	Normal	3.5–10 x10^3/uL
RBC	5.27	Normal	3.5–5.5 x10^6/uL
HGB	16	Normal	12–16 g/dl
PLT	263	Normal	150–450 x10^3/uL

Management

The patient was admitted to the hospital and started on the following: 

1) sodium valproate 500 mg po bid; 2) risperidone 2 mg po bid; and 3) promethazine 25mg pm for 1 week and then prn for agitation and insomnia. The patient was enrolled in both individual and group psychotherapeutic sessions, and he participated in a few social and spiritual counseling sessions. The latter focused on addressing his drug consumption patterns and associated consequences.

Course and follow-up

The patient was admitted and started on the above medications, and he showed some improvement; later, he was discharged and was followed up through the outpatient department OPD. Further assessment confirmed the presence of substance abuse of hash and stimulants, including amphetamine. A urine drug screening later came out positive.

**Figure 1:**
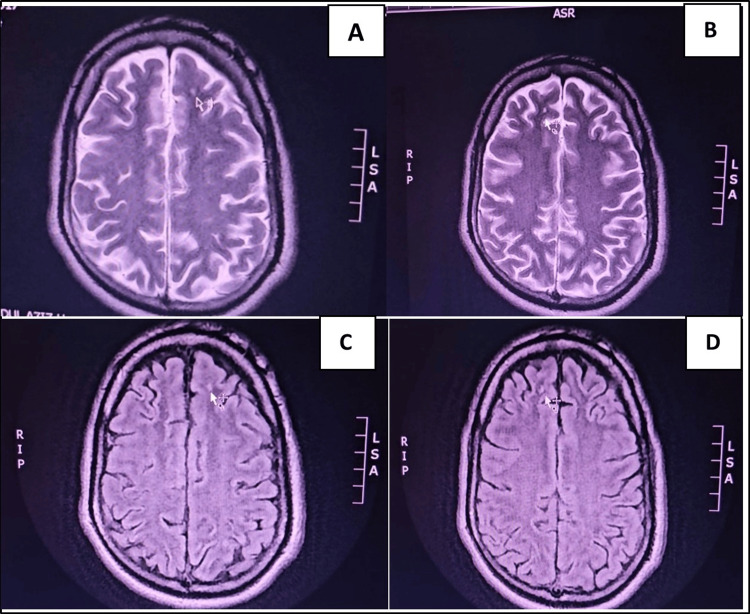
Axial brain MRI images showing frontal changes Axial brain MRI images; T2-weighted. A, B and axial FLAIR C, D, showing Rt. and Lt. frontal subcortical small hyperintense areas (arrows).

**Figure 2:**
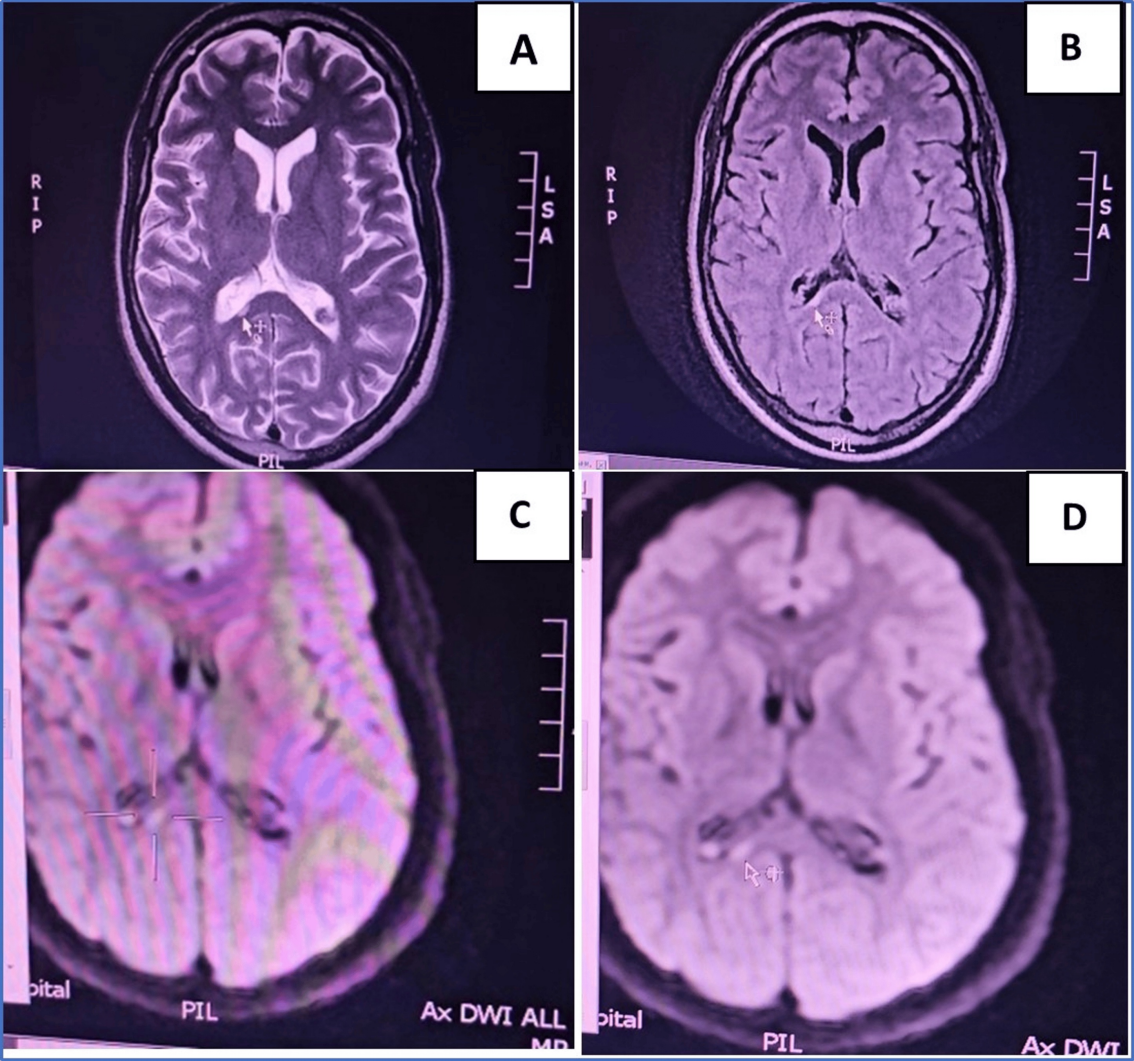
Axial brain MRI images showing corpus callosum splenium changes Axial brain MRI images; T2-weighted: A, axial FLAIR: B, and DWI: C, D Showing Rt. corpus callosum splenium small hyperintense area (arrows).

The patient was not regular in his appointments at the outpatient clinic. In the last year, the patient was admitted for reevaluation because he still had the same mood and psychotic features associated with poor attention and concentration. His father stated that he has been ill for the last 10 years and has never returned to normal. The treating doctors noticed a scar on his head, and after repeated questions, the patient declared a history of a car accident 10 years earlier, resulting in a head injury and loss of consciousness. There was a significant loss of consciousness for about one month when he was kept in the Intensive Care Unit (ICU) in a local general hospital. An MRI was requested by the treating physician, and the result showed significant changes in the corpus callosum and frontal lobe (Table 2, Figures 1, 2). The patient’s diagnosis was changed to 1) bipolar disorder with psychotic features due to traumatic brain injury (frontal lobe) and 2) amphetamine and hash use disorder.

**Table 2:** MRI brain report

Exams	Non-contrast MRI brain
Clinical information	A 28-year-old male with a history of old head trauma with disturbed behavior and psychosis
Technique of the examination	Multiplanar as well as multi-sequential images
Findings	- Bilateral frontal subcortical and corpus callosum splenium right-side multiple scattered small T2-hyperintensities with no apparent restricted diffusion and no edema or mass effect - Normal parenchymal signal of the brain stem and cerebellum - No evidence of recent infarction or hemorrhage - No extra-axial or extra-axial mass - No midline shift - Normal size and configuration of the ventricular system - No evidence of hydrocephalus or brain atrophy
Conclusion	Bilateral frontal multiple subcortical and corpus callosum nonspecific T2-hyperintensities as described, for clinical correlation and follow-up

The valproate dose was increased to 1500 mg/day divided, and risperidone to 3 mgpo bid. The patient showed a better response to treatment, with a delayed improvement in cognitive features. He continued to be in a state of partial remission, and it remains uncertain whether he had ceased his drug usage by the time of this report.

## Discussion

The mental health consequences of TBI are complex and varied, including personality changes, affective symptoms, and the worsening or new onset of psychiatric disorders. Even mild TBI is associated with affective symptoms and psychiatric disorders [6].

Mood disorders are among the most common consequences of traumatic brain injury (TBI). The most common mood disorders after TBI are major depressive disorder and anxiety disorders, with prevalence ranges of 13% to 53% and 11% to 70%, respectively [7].

Bipolar and related disorders are less frequent after TBI, with prevalence rates like those in the general population. Rates of up to 1% to 9% have been reported in some early TBI studies [8,9]. In this patient, the head trauma was severe, and the main psychiatric features were manic with psychotic features accompanied by frontal lobe symptoms.

Frontal lobe lesions can result in disturbances of executive functions, personality changes, emotional disturbances, impaired motor function, language issues, and impaired judgment (frontal lobe syndrome). This area is crucial for many higher cognitive functions, including problem-solving, planning, memory, and controlling behavior and emotions. Frontal lobe syndrome can result from various causes, including traumatic brain injury, stroke, tumors, degenerative diseases (like dementia), or infections. Treatment and management depend on the underlying cause and the specific symptoms experienced by the individual.

TBI impacts frontal lobe functions significantly, and the consequences of TBI are dynamic, with unique characteristics expressed over the lifespan. There is a growing consensus that the outcome after TBI should be understood as a dynamic process with individual trajectories [10]. A study was done on all Danes born between 1977 and 2000, including 1.4 million people. They were followed up until 2010. Of the study sample, 113,906 went to the hospital for a head injury. Four percent of those individuals were subsequently determined to have a mental illness. The likelihood of acquiring a mental illness is most prominent within the initial year following a cranial trauma; however, it remains elevated even after a span of 15 years. People with TBI are 1.28 times more likely to have bipolar disorder. This is especially true if the head injury happened between the ages of 11 and 15. It is believed that TBI causes brain inflammation, which can lead to mental health issues [11].

Mania symptoms after TBI can be characterized by disinhibition, impulsivity, aggression, mood lability, and reduced executive functioning [12]. TBI-related mania may also be associated with right-sided brain lesions, particularly in the frontal lobe [13]. Although TBI can increase the chances of developing bipolar disorder and can lead to cognitive impairment, emotional difficulties, and behavioral changes [14], it can be difficult for clinicians to decide whether the prevailing diagnosis is the primary mental illness or the medical problem causing the mental illness, in this case, the TBI.

The relationship between age and the prevalence of bipolar disorder after traumatic brain injury (TBI) is a complex topic. Although specific age-related prevalence data is not readily available, some relevant insights can be gathered from existing research. In a cross-sectional study of 505 patients with bipolar disorder, of whom 37 (7.3%) reported a premorbid TBI, the mean age at the incidence of TBI was 10 years, and the mean duration from TBI to the onset of bipolar disorder was approximately 8.9 years. This study indicates that TBI earlier in life may be associated with the later development of bipolar disorder, although it doesn’t provide a direct comparison of bipolar disorder prevalence across different age groups after TBI [15].

For our patient, the onset of head injury and TBI occurred at the age of 18, which roughly aligns with the result of the previous study. Furthermore, in a study in Taiwan, investigators used a health insurance database to compare ICD-9 diagnoses of mood disorders between patients who had two outpatient claims or one inpatient claim for TBI in 2000-2004 and age- and sex-matched controls with non-TBI-related claims in the same period. They found that TBI increases the risk of mood disorders in adolescents [16]. The brain areas affected by the TBI, as visualized by MRI, are variable. TBI-related brain damage often involves areas like the frontal cortex, basal ganglia, and temporal lobes, which are crucial in mood regulation [17].

In this study, the patient had multiple scattered small hyperintensities in the corpus callosum and frontal subcortex. Brain imaging is important in diagnosing TBI, and physicians, particularly psychiatrists, need to have a high index of suspicion for organic causes of psychiatric symptom presentations, especially new ones, so they should use available neuroimaging facilities to augment the conclusion that the medical problem is behind the psychiatric presentations. Moschopoulos and others reported a case of a 74-year-old man who developed behavioral disturbances and was diagnosed with mania following a traumatic brain injury. Brain imaging revealed a sizable gliosis in the right frontal lobe, and the patient developed severe cognitive decline [18]. Mania can occur with injury to either hemisphere, as in our patient, but it is primarily associated with right-side frontal lobe injury, as shown in a case report of an adult patient with fetal alcohol syndrome who had manic behavior resulting from a left frontal closed head injury [19]. These MRI changes might not be specific, but they need to be correlated with the clinical picture.

Injury to the corpus callosum from TBI typically occurs as a result of axonal injury, impacting well-structured white matter pathways. It is important that we also take into consideration the potential effects of substance abuse. The prognosis and the course of recovery from TBI are variable, depending not only on the specifics of the injury but also on pre-injury and post-injury factors. These factors may favor or impede recovery and include age, prior history of TBI, neurological, psychiatric, and substance use comorbidities and complications, genetics, the timeliness and effectiveness of medical and rehabilitative interventions, and psychosocial support [5].

Our patient had suffered a severe injury and continued to have a chronic, unremitting course with some resistance to treatment and no full recovery. The neurocognitive impairments and associated functional limitations produced by moderate and severe TBI typically improve over weeks to months after the injury, although long-term neurocognitive recovery is often incomplete among individuals with more severe injuries.

Nonetheless, neurocognitive and functional improvement may continue for years after moderate or severe TBI, with more individuals cognitively improving than declining during the first five years post injury. Moderate and severe TBI have been associated with an increased risk of depression, aggression, and possibly neurodegenerative diseases such as Alzheimer’s disease and frontotemporal degeneration [5].

The management of mood disorders after TBI includes pharmacologic and nonpharmacologic interventions, and both are commonly used for treatment. Pharmacological agents are effective in treating post-TBI mood disorders. These interventions aim to improve processing speed, working memory, visual processing, and compensatory measures for memory loss. Psychosocial rehabilitation plays a crucial role in the management of mood disorders after TBI. It involves the rehabilitation of vocation and education and the building of social support [7]. The patient in our study had shown a somewhat difficult treatment response and long-suffering cognitive and behavioral symptoms. These were possibly aggravated by his drug use over the long period of his illness. Substances can further impair cognitive function in TBI patients, leading to problems with memory, attention, reasoning, and emotional regulation. Stimulants, in particular, can exacerbate anxiety, agitation, and insomnia in individuals with TBI. Substances can exacerbate existing psychiatric features, making them more severe and clearly indistinguishable from TBI consequences.

Koparal and Coşar reported a case of secondary mania following a traumatic brain injury. The patient experienced a difficult response to treatment as well as recurrence and a long disease and treatment period [20]. Management of depression and bipolar disorders in patients with a history of TBI largely follows the same standard treatment recommendations as for neurologically intact patient populations, except for a greater focus on tolerability and safety [9].

Currently, treatment options are often dictated by expert opinion rather than by rigorous, adequately designed, and sufficiently large studies [8]. There are no specific guidelines for treating these patients; rather, there is a need for additional drug trials to establish effective treatments.

## Conclusions

I describe an uncommon case of bipolar disorder due to a traumatic brain injury diagnosed after 10 years of being misdiagnosed as a schizoaffective disorder. Unveiling the car accident history suddenly raised concerns about this patient, not only regarding the diagnosis but also the management strategies. This case highlights the importance of early diagnosis of medical conditions presenting with psychiatric symptoms, because even after a long time of suffering, the clue might be a head trauma discovered suddenly. The hint for this case was the longitudinal and unrecovered course and poor response to the usual dose of psychiatric medications. It is important to implement the psychosocial modalities of management in such cases in addition to biological therapies, and comorbidities like substance use disorders should also be considered.
