# Correction: Bipolar Disorder Due to Traumatic Brain Injury: A Case Report

**DOI:** 10.7759/cureus.c218

**Published:** 2025-05-01

**Authors:** Yasir Altuwairqi

**Affiliations:** 1 Medicine/Psychiatry, Taif University, Taif, SAU

It was incorrectly stated in the Discussion section that people with TBI are 28 times more likely to have bipolar disorder. This was a typo and has been corrected to state that people with TBI are 1.28 times more likely to have bipolar disorder.

